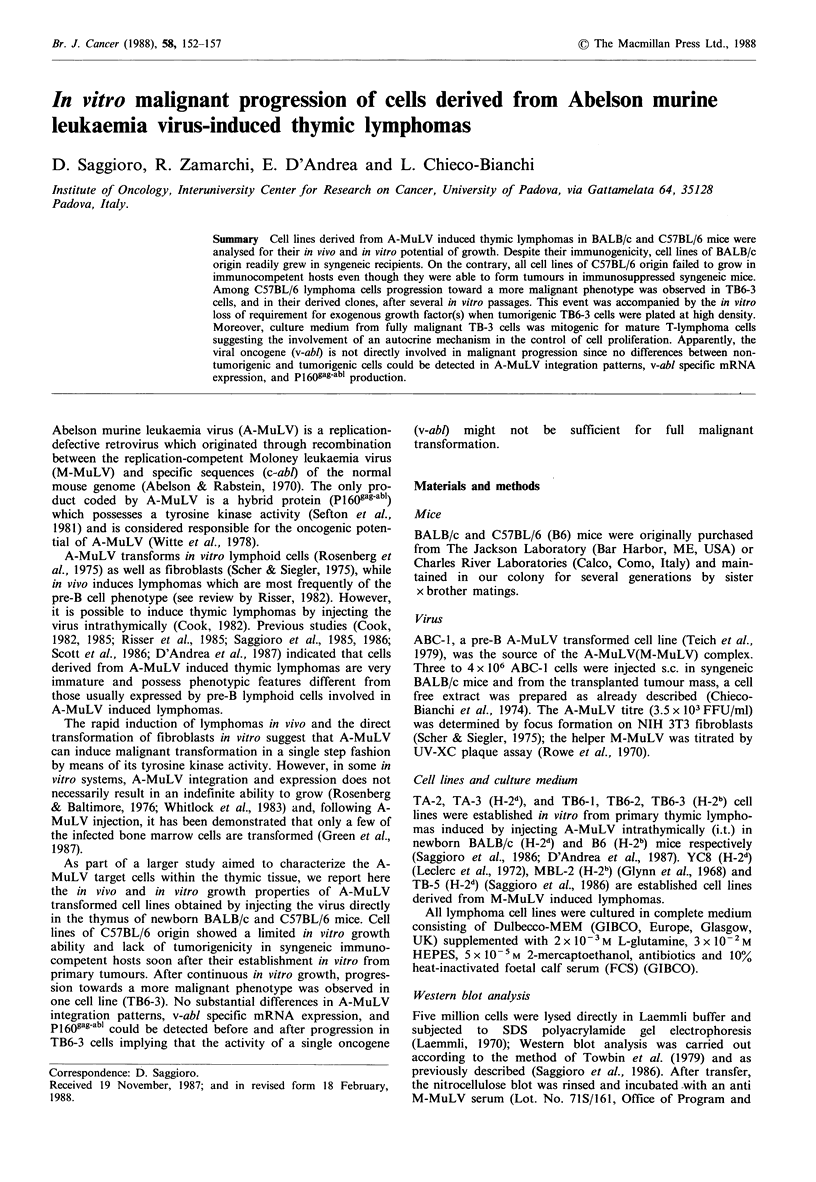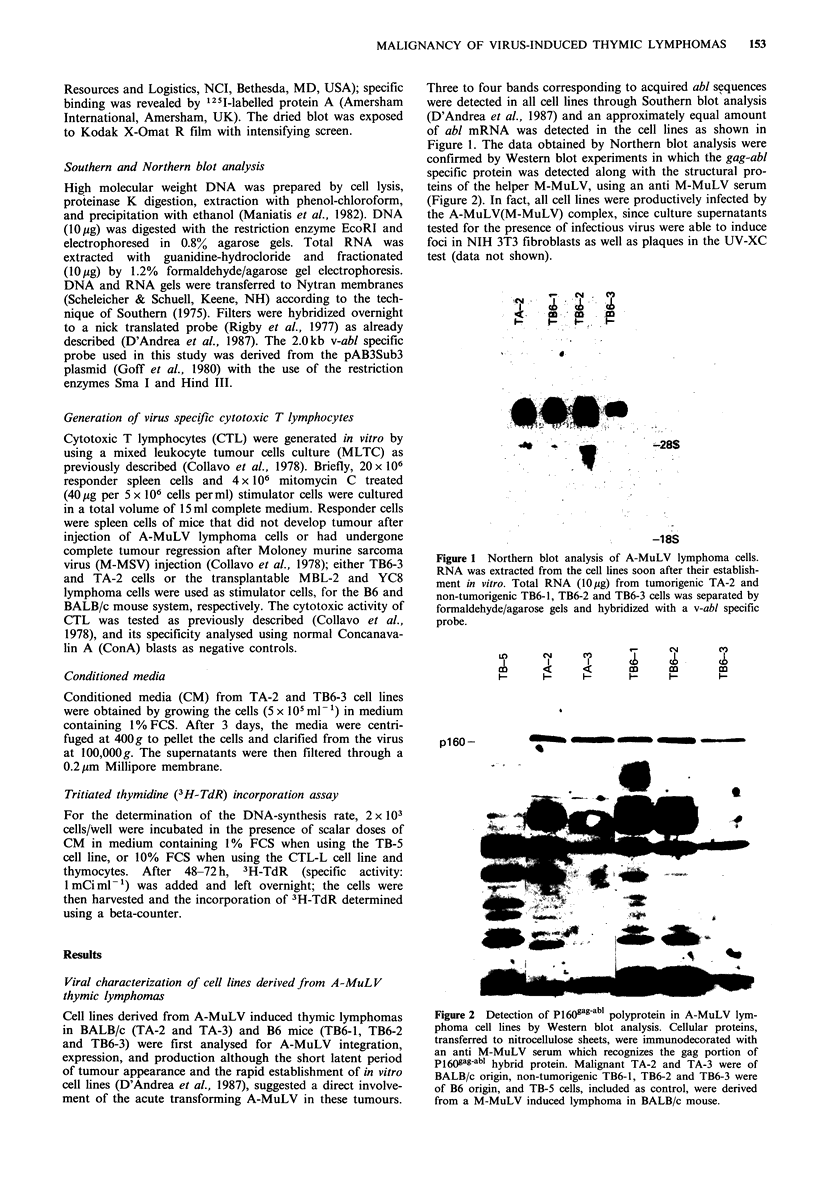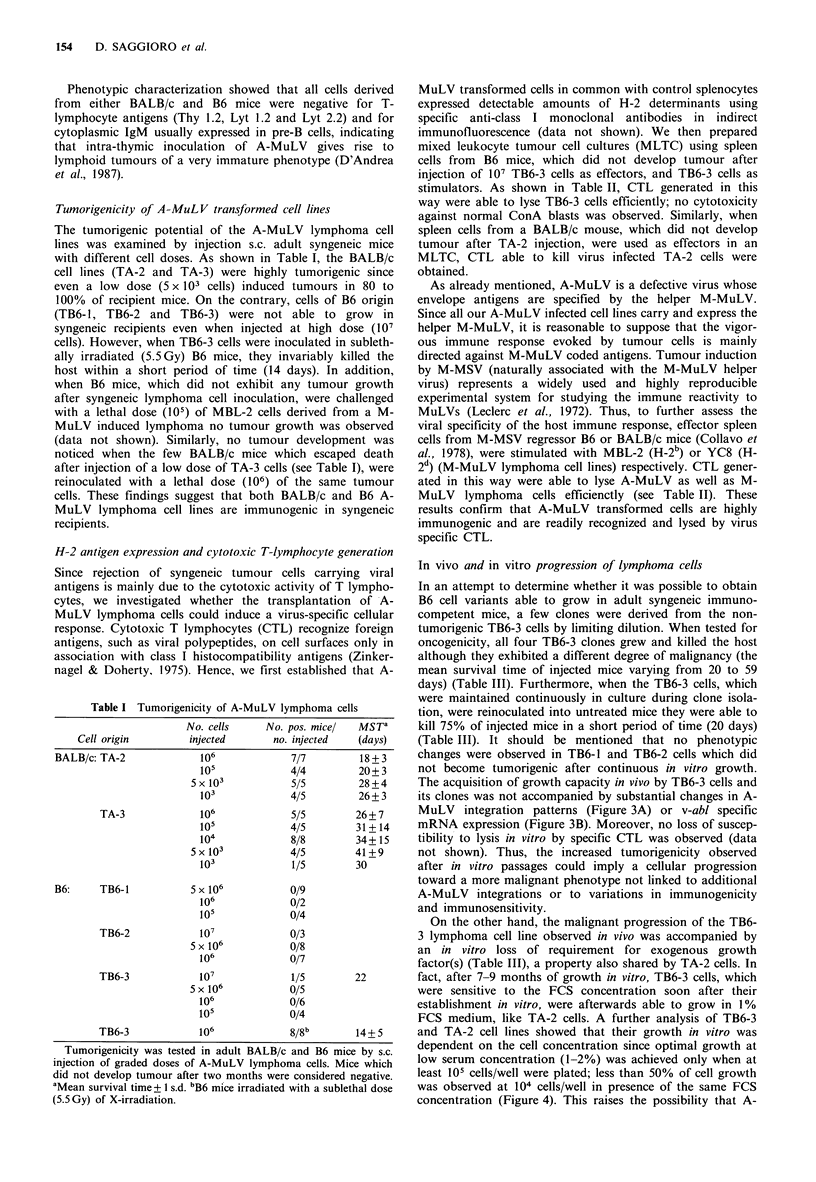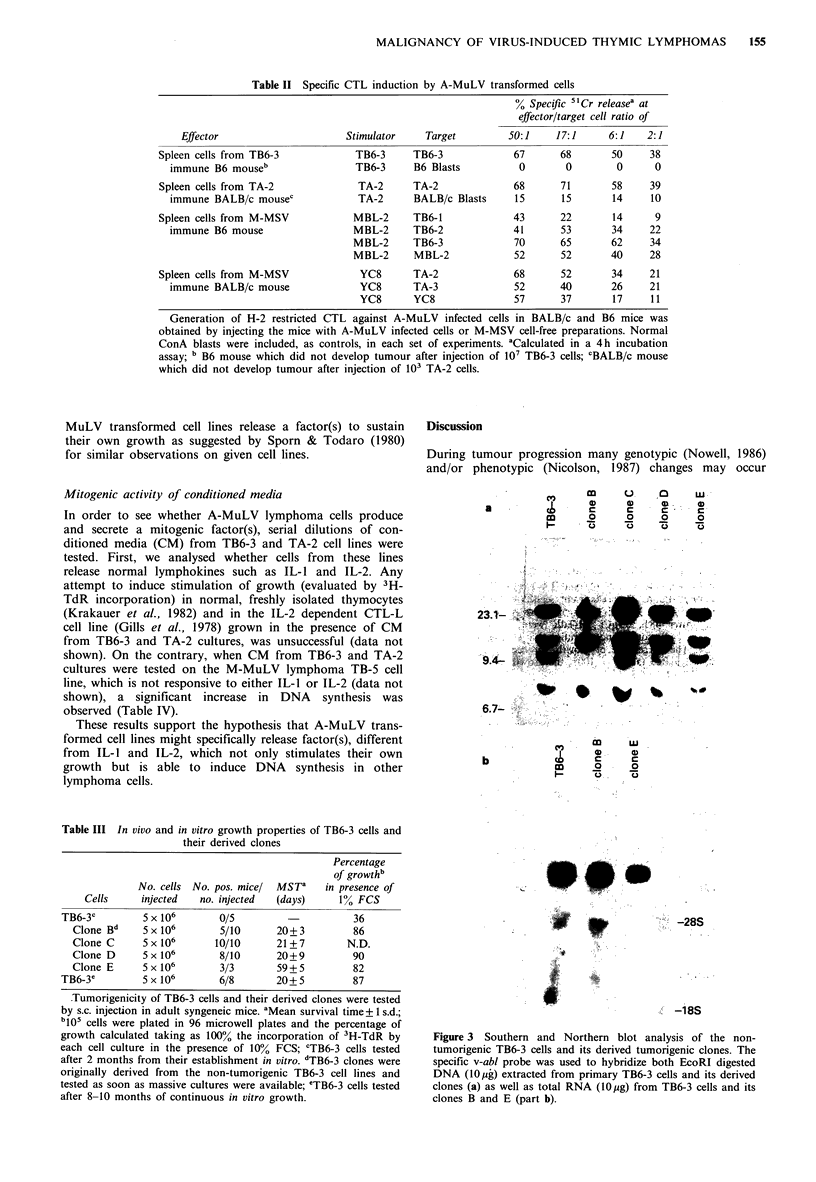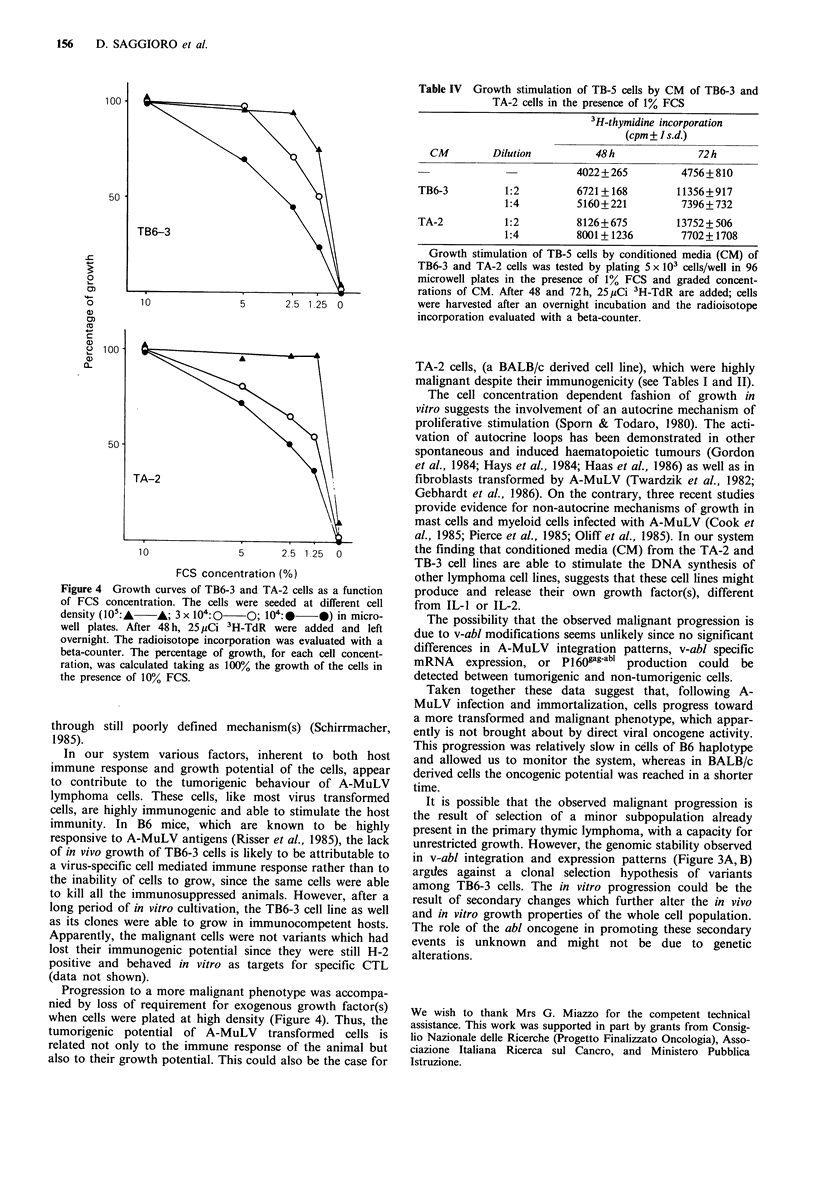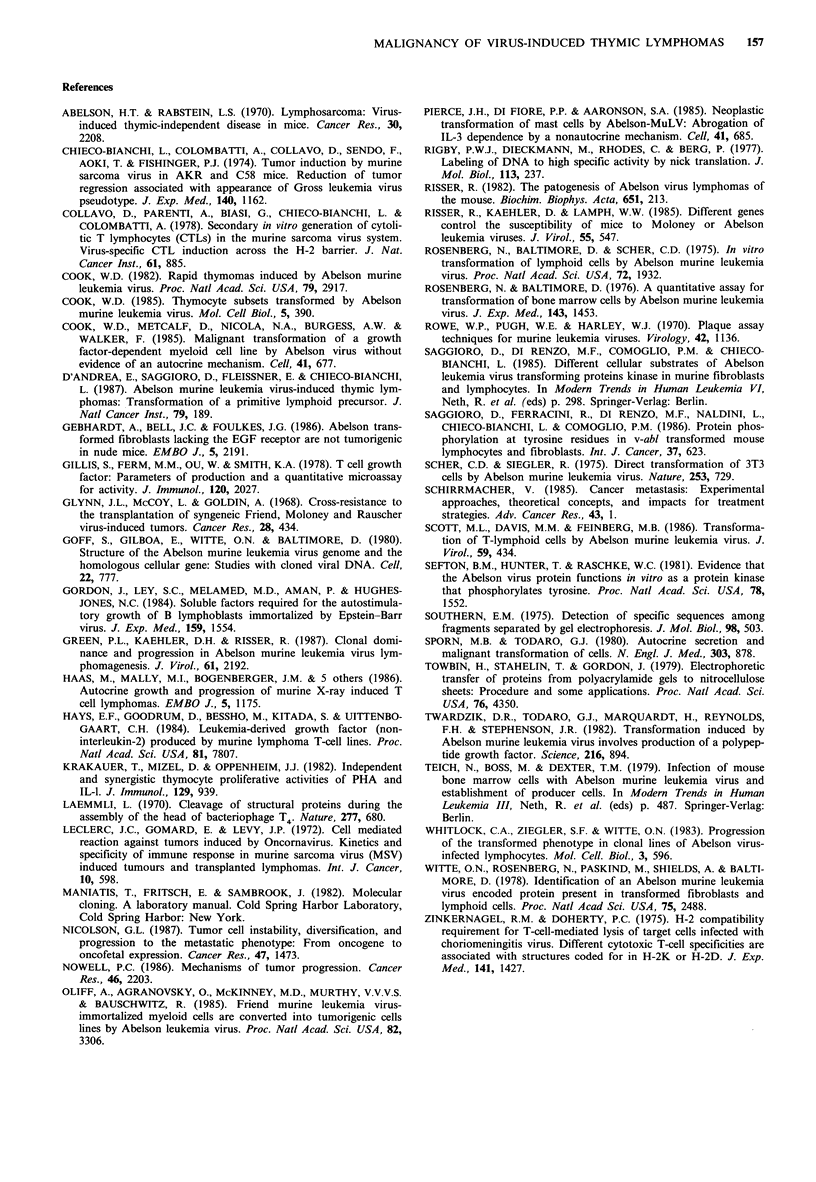# In vitro malignant progression of cells derived from Abelson murine leukaemia virus-induced thymic lymphomas.

**DOI:** 10.1038/bjc.1988.183

**Published:** 1988-08

**Authors:** D. Saggioro, R. Zamarchi, E. D'Andrea, L. Chieco-Bianchi

**Affiliations:** Institute of Oncology, University of Padova, Italy.

## Abstract

**Images:**


*Br. J. Cancer* (1988), **58**, 152–157 © The Macmillan Press Ltd., 1988

### *In vitro* malignant progression of cells derived from Abelson murine leukaemia virus-induced thymic lymphomas

D. Saggioro, R. Zamarchi, E. D'Andrea and L. Chieco-Bianchi

*Institute of Oncology, Interuniversity Center for Research on Cancer, University of Padova, via Gattamelata 64, 35128 Padova, Italy.*

**Summary** Cell lines derived from A-MuLV induced thymic lymphomas in BALB/c and C57BL/6 mice were analysed for their *in vivo* and *in vitro* potential of growth. Despite their immunogenicity, cell lines of BALB/c origin readily grew in syngeneic recipients. On the contrary, all cell lines of C57BL/6 origin failed to grow in immunocompetent hosts even though they were able to form tumours in immunosuppressed syngeneic mice. Among C57BL/6 lymphoma cells progression toward a more malignant phenotype was observed in TB6-3 cells, and in their derived clones, after several *in vitro* passages. This event was accompanied by the *in vitro* loss of requirement for exogenous growth factor(s) when tumorigenic TB6-3 cells were plated at high density. Moreover, culture medium from fully malignant TB-3 cells was mitogenic for mature T-lymphoma cells suggesting the involvement of an autocrine mechanism in the control of cell proliferation. Apparently, the viral oncogene (v-*abl*) is not directly involved in malignant progression since no differences between non-tumorigenic and tumorigenic cells could be detected in A-MuLV integration patterns, v-*abl* specific mRNA expression, and P160$^{gag\text{-}abl}$ production.

Abelson murine leukaemia virus (A-MuLV) is a replication-defective retrovirus which originated through recombination between the replication-competent Moloney leukaemia virus (M-MuLV) and specific sequences (c-*abl*) of the normal mouse genome (Abelson & Rabstein, 1970). The only product coded by A-MuLV is a hybrid protein (P160$^{gag\text{-}abl}$) which possesses a tyrosine kinase activity (Sefton *et al.*, 1981) and is considered responsible for the oncogenic potential of A-MuLV (Witte *et al.*, 1978).

A-MuLV transforms *in vitro* lymphoid cells (Rosenberg *et al.*, 1975) as well as fibroblasts (Scher & Siegler, 1975), while *in vivo* induces lymphomas which are most frequently of the pre-B cell phenotype (see review by Risser, 1982). However, it is possible to induce thymic lymphomas by injecting the virus intrathymically (Cook, 1982). Previous studies (Cook, 1982, 1985; Risser *et al.*, 1985; Saggioro *et al.*, 1985, 1986; Scott *et al.*, 1986; D'Andrea *et al.*, 1987) indicated that cells derived from A-MuLV induced thymic lymphomas are very immature and possess phenotypic features different from those usually expressed by pre-B lymphoid cells involved in A-MuLV induced lymphomas.

The rapid induction of lymphomas *in vivo* and the direct transformation of fibroblasts *in vitro* suggest that A-MuLV can induce malignant transformation in a single step fashion by means of its tyrosine kinase activity. However, in some *in vitro* systems, A-MuLV integration and expression does not necessarily result in an indefinite ability to grow (Rosenberg & Baltimore, 1976; Whitlock *et al.*, 1983) and, following A-MuLV injection, it has been demonstrated that only a few of the infected bone marrow cells are transformed (Green *et al.*, 1987).

As part of a larger study aimed to characterize the A-MuLV target cells within the thymic tissue, we report here the *in vivo* and *in vitro* growth properties of A-MuLV transformed cell lines obtained by injecting the virus directly in the thymus of newborn BALB/c and C57BL/6 mice. Cell lines of C57BL/6 origin showed a limited *in vitro* growth ability and lack of tumorigenicity in syngeneic immunocompetent hosts soon after their establishment *in vitro* from primary tumours. After continuous *in vitro* growth, progression towards a more malignant phenotype was observed in one cell line (TB6-3). No substantial differences in A-MuLV integration patterns, v-*abl* specific mRNA expression, and P160$^{gag\text{-}abl}$ could be detected before and after progression in TB6-3 cells implying that the activity of a single oncogene (v-*abl*) might not be sufficient for full malignant transformation.

Correspondence: D. Saggioro.
Received 19 November, 1987; and in revised form 18 February, 1988.

### Materials and methods

### *Mice*

BALB/c and C57BL/6 (B6) mice were originally purchased from The Jackson Laboratory (Bar Harbor, ME, USA) or Charles River Laboratories (Calco, Como, Italy) and maintained in our colony for several generations by sister × brother matings.

### *Virus*

ABC-1, a pre-B A-MuLV transformed cell line (Teich *et al.*, 1979), was the source of the A-MuLV(M-MuLV) complex. Three to $4 \times 10^6$ ABC-1 cells were injected s.c. in syngeneic BALB/c mice and from the transplanted tumour mass, a cell free extract was prepared as already described (Chieco-Bianchi *et al.*, 1974). The A-MuLV titre ($3.5 \times 10^3$ FFU/ml) was determined by focus formation on NIH 3T3 fibroblasts (Scher & Siegler, 1975); the helper M-MuLV was titrated by UV-XC plaque assay (Rowe *et al.*, 1970).

### *Cell lines and culture medium*

TA-2, TA-3 (H-2$^d$), and TB6-1, TB6-2, TB6-3 (H-2$^b$) cell lines were established *in vitro* from primary thymic lymphomas induced by injecting A-MuLV intrathymically (i.t.) in newborn BALB/c (H-2$^d$) and B6 (H-2$^b$) mice respectively (Saggioro *et al.*, 1986; D'Andrea *et al.*, 1987). YC8 (H-2$^d$) (Leclerc *et al.*, 1972), MBL-2 (H-2$^b$) (Glynn *et al.*, 1968) and TB-5 (H-2$^d$) (Saggioro *et al.*, 1986) are established cell lines derived from M-MuLV induced lymphomas.

All lymphoma cell lines were cultured in complete medium consisting of Dulbecco-MEM (GIBCO, Europe, Glasgow, UK) supplemented with $2 \times 10^{-3}$ M L-glutamine, $3 \times 10^{-2}$ M HEPES, $5 \times 10^{-5}$ M 2-mercaptoethanol, antibiotics and 10% heat-inactivated foetal calf serum (FCS) (GIBCO).

### *Western blot analysis*

Five million cells were lysed directly in Laemmli buffer and subjected to SDS polyacrylamide gel electrophoresis (Laemmli, 1970); Western blot analysis was carried out according to the method of Towbin *et al.* (1979) and as previously described (Saggioro *et al.*, 1986). After transfer, the nitrocellulose blot was rinsed and incubated with an anti M-MuLV serum (Lot. No. 71S/161, Office of Program and

Resources and Logistics, NCI, Bethesda, MD, USA); specific binding was revealed by $^{125}$I-labelled protein A (Amersham International, Amersham, UK). The dried blot was exposed to Kodak X-Omat R film with intensifying screen.

*Southern and Northern blot analysis*

High molecular weight DNA was prepared by cell lysis, proteinase K digestion, extraction with phenol-chloroform, and precipitation with ethanol (Maniatis *et al.*, 1982). DNA (10 $\mu$g) was digested with the restriction enzyme EcoRI and electrophoresed in 0.8% agarose gels. Total RNA was extracted with guanidine-hydrocloride and fractionated (10 $\mu$g) by 1.2% formaldehyde/agarose gel electrophoresis. DNA and RNA gels were transferred to Nytran membranes (Scheleicher & Schuell, Keene, NH) according to the technique of Southern (1975). Filters were hybridized overnight to a nick translated probe (Rigby *et al.*, 1977) as already described (D'Andrea *et al.*, 1987). The 2.0 kb v-*abl* specific probe used in this study was derived from the pAB3Sub3 plasmid (Goff *et al.*, 1980) with the use of the restriction enzymes Sma I and Hind III.

*Generation of virus specific cytotoxic T lymphocytes*

Cytotoxic T lymphocytes (CTL) were generated *in vitro* by using a mixed leukocyte tumour cells culture (MLTC) as previously described (Collavo *et al.*, 1978). Briefly, $20 \times 10^6$ responder spleen cells and $4 \times 10^6$ mitomycin C treated (40 $\mu$g per $5 \times 10^6$ cells per ml) stimulator cells were cultured in a total volume of 15 ml complete medium. Responder cells were spleen cells of mice that did not develop tumour after injection of A-MuLV lymphoma cells or had undergone complete tumour regression after Moloney murine sarcoma virus (M-MSV) injection (Collavo *et al.*, 1978); either TB6-3 and TA-2 cells or the transplantable MBL-2 and YC8 lymphoma cells were used as stimulator cells, for the B6 and BALB/c mouse system, respectively. The cytotoxic activity of CTL was tested as previously described (Collavo *et al.*, 1978), and its specificity analysed using normal Concanavalin A (ConA) blasts as negative controls.

*Conditioned media*

Conditioned media (CM) from TA-2 and TB6-3 cell lines were obtained by growing the cells ($5 \times 10^5$ ml$^{-1}$) in medium containing 1% FCS. After 3 days, the media were centrifuged at 400 *g* to pellet the cells and clarified from the virus at 100,000 *g*. The supernatants were then filtered through a 0.2 $\mu$m Millipore membrane.

*Tritiated thymidine ($^3$H-TdR) incorporation assay*

For the determination of the DNA-synthesis rate, $2 \times 10^3$ cells/well were incubated in the presence of scalar doses of CM in medium containing 1% FCS when using the TB-5 cell line, or 10% FCS when using the CTL-L cell line and thymocytes. After 48–72 h, $^3$H-TdR (specific activity: 1 mCi ml$^{-1}$) was added and left overnight; the cells were then harvested and the incorporation of $^3$H-TdR determined using a beta-counter.

### Results

*Viral characterization of cell lines derived from A-MuLV thymic lymphomas*

Cell lines derived from A-MuLV induced thymic lymphomas in BALB/c (TA-2 and TA-3) and B6 mice (TB6-1, TB6-2 and TB6-3) were first analysed for A-MuLV integration, expression, and production although the short latent period of tumour appearance and the rapid establishment of *in vitro* cell lines (D'Andrea *et al.*, 1987), suggested a direct involvement of the acute transforming A-MuLV in these tumours. Three to four bands corresponding to acquired *abl* sequences were detected in all cell lines through Southern blot analysis (D'Andrea *et al.*, 1987) and an approximately equal amount of *abl* mRNA was detected in the cell lines as shown in Figure 1. The data obtained by Northern blot analysis were confirmed by Western blot experiments in which the *gag-abl* specific protein was detected along with the structural proteins of the helper M-MuLV, using an anti M-MuLV serum (Figure 2). In fact, all cell lines were productively infected by the A-MuLV(M-MuLV) complex, since culture supernatants tested for the presence of infectious virus were able to induce foci in NIH 3T3 fibroblasts as well as plaques in the UV-XC test (data not shown).

**Figure 1** Northern blot analysis of A-MuLV lymphoma cells. RNA was extracted from the cell lines soon after their establishment *in vitro*. Total RNA (10 $\mu$g) from tumorigenic TA-2 and non-tumorigenic TB6-1, TB6-2 and TB6-3 cells was separated by formaldehyde/agarose gels and hybridized with a v-*abl* specific probe.

**Figure 2** Detection of P160$^{gag\text{-}abl}$ polyprotein in A-MuLV lymphoma cell lines by Western blot analysis. Cellular proteins, transferred to nitrocellulose sheets, were immunodecorated with an anti M-MuLV serum which recognizes the gag portion of P160$^{gag\text{-}abl}$ hybrid protein. Malignant TA-2 and TA-3 were of BALB/c origin, non-tumorigenic TB6-1, TB6-2 and TB6-3 were of B6 origin, and TB-5 cells, included as control, were derived from a M-MuLV induced lymphoma in BALB/c mouse.

Phenotypic characterization showed that all cells derived from either BALB/c and B6 mice were negative for T-lymphocyte antigens (Thy 1.2, Lyt 1.2 and Lyt 2.2) and for cytoplasmic IgM usually expressed in pre-B cells, indicating that intra-thymic inoculation of A-MuLV gives rise to lymphoid tumours of a very immature phenotype (D'Andrea *et al.*, 1987).

*Tumorigenicity of A-MuLV transformed cell lines*

The tumorigenic potential of the A-MuLV lymphoma cell lines was examined by injection s.c. adult syngeneic mice with different cell doses. As shown in Table I, the BALB/c cell lines (TA-2 and TA-3) were highly tumorigenic since even a low dose ($5\times10^3$ cells) induced tumours in 80 to 100% of recipient mice. On the contrary, cells of B6 origin (TB6-1, TB6-2 and TB6-3) were not able to grow in syngeneic recipients even when injected at high dose ($10^7$ cells). However, when TB6-3 cells were inoculated in sublethally irradiated (5.5 Gy) B6 mice, they invariably killed the host within a short period of time (14 days). In addition, when B6 mice, which did not exhibit any tumour growth after syngeneic lymphoma cell inoculation, were challenged with a lethal dose ($10^5$) of MBL-2 cells derived from a M-MuLV induced lymphoma no tumour growth was observed (data not shown). Similarly, no tumour development was noticed when the few BALB/c mice which escaped death after injection of a low dose of TA-3 cells (see Table I), were reinoculated with a lethal dose ($10^6$) of the same tumour cells. These findings suggest that both BALB/c and B6 A-MuLV lymphoma cell lines are immunogenic in syngeneic recipients.

*H-2 antigen expression and cytotoxic T-lymphocyte generation*

Since rejection of syngeneic tumour cells carrying viral antigens is mainly due to the cytotoxic activity of T lymphocytes, we investigated whether the transplantation of A-MuLV lymphoma cells could induce a virus-specific cellular response. Cytotoxic T lymphocytes (CTL) recognize foreign antigens, such as viral polypeptides, on cell surfaces only in association with class I histocompatibility antigens (Zinkernagel & Doherty, 1975). Hence, we first established that A-MuLV transformed cells in common with control splenocytes expressed detectable amounts of H-2 determinants using specific anti-class I monoclonal antibodies in indirect immunofluorescence (data not shown). We then prepared mixed leukocyte tumour cell cultures (MLTC) using spleen cells from B6 mice, which did not develop tumour after injection of $10^7$ TB6-3 cells as effectors, and TB6-3 cells as stimulators. As shown in Table II, CTL generated in this way were able to lyse TB6-3 cells efficiently; no cytotoxicity against normal ConA blasts was observed. Similarly, when spleen cells from a BALB/c mouse, which did not develop tumour after TA-2 injection, were used as effectors in an MLTC, CTL able to kill virus infected TA-2 cells were obtained.

As already mentioned, A-MuLV is a defective virus whose envelope antigens are specified by the helper M-MuLV. Since all our A-MuLV infected cell lines carry and express the helper M-MuLV, it is reasonable to suppose that the vigorous immune response evoked by tumour cells is mainly directed against M-MuLV coded antigens. Tumour induction by M-MSV (naturally associated with the M-MuLV helper virus) represents a widely used and highly reproducible experimental system for studying the immune reactivity to MuLVs (Leclerc *et al.*, 1972). Thus, to further assess the viral specificity of the host immune response, effector spleen cells from M-MSV regressor B6 or BALB/c mice (Collavo *et al.*, 1978), were stimulated with MBL-2 (H-$2^b$) or YC8 (H-$2^d$) (M-MuLV lymphoma cell lines) respectively. CTL generated in this way were able to lyse A-MuLV as well as M-MuLV lymphoma cells efficienctly (see Table II). These results confirm that A-MuLV transformed cells are highly immunogenic and are readily recognized and lysed by virus specific CTL.

**Table I** Tumorigenicity of A-MuLV lymphoma cells

| Cell origin | | *No. cells injected* | *No. pos. mice/ no. injected* | *MST*[a] *(days)* |
|---|---|---|---|---|
| BALB/c: | TA-2 | $10^6$ | 7/7 | $18\pm3$ |
| | | $10^5$ | 4/4 | $20\pm3$ |
| | | $5\times10^3$ | 5/5 | $28\pm4$ |
| | | $10^3$ | 4/5 | $26\pm3$ |
| | TA-3 | $10^6$ | 5/5 | $26\pm7$ |
| | | $10^5$ | 4/5 | $31\pm14$ |
| | | $10^4$ | 8/8 | $34\pm15$ |
| | | $5\times10^3$ | 4/5 | $41\pm9$ |
| | | $10^3$ | 1/5 | 30 |
| B6: | TB6-1 | $5\times10^6$ | 0/9 | |
| | | $10^6$ | 0/2 | |
| | | $10^5$ | 0/4 | |
| | TB6-2 | $10^7$ | 0/3 | |
| | | $5\times10^6$ | 0/8 | |
| | | $10^6$ | 0/7 | |
| | TB6-3 | $10^7$ | 1/5 | 22 |
| | | $5\times10^6$ | 0/5 | |
| | | $10^6$ | 0/6 | |
| | | $10^5$ | 0/4 | |
| | TB6-3 | $10^6$ | 8/8[b] | $14\pm5$ |

Tumorigenicity was tested in adult BALB/c and B6 mice by s.c. injection of graded doses of A-MuLV lymphoma cells. Mice which did not develop tumour after two months were considered negative. [a]Mean survival time $\pm$ 1 s.d. [b]B6 mice irradiated with a sublethal dose (5.5 Gy) of X-irradiation.

*In vivo and in vitro progression of lymphoma cells*

In an attempt to determine whether it was possible to obtain B6 cell variants able to grow in adult syngeneic immunocompetent mice, a few clones were derived from the non-tumorigenic TB6-3 cells by limiting dilution. When tested for oncogenicity, all four TB6-3 clones grew and killed the host although they exhibited a different degree of malignancy (the mean survival time of injected mice varying from 20 to 59 days) (Table III). Furthermore, when the TB6-3 cells, which were maintained continuously in culture during clone isolation, were reinoculated into untreated mice they were able to kill 75% of injected mice in a short period of time (20 days) (Table III). It should be mentioned that no phenotypic changes were observed in TB6-1 and TB6-2 cells which did not become tumorigenic after continuous *in vitro* growth. The acquisition of growth capacity *in vivo* by TB6-3 cells and its clones was not accompanied by substantial changes in A-MuLV integration patterns (Figure 3A) or v-*abl* specific mRNA expression (Figure 3B). Moreover, no loss of susceptibility to lysis *in vitro* by specific CTL was observed (data not shown). Thus, the increased tumorigenicity observed after *in vitro* passages could imply a cellular progression toward a more malignant phenotype not linked to additional A-MuLV integrations or to variations in immunogenicity and immunosensitivity.

On the other hand, the malignant progression of the TB6-3 lymphoma cell line observed *in vivo* was accompanied by an *in vitro* loss of requirement for exogenous growth factor(s) (Table III), a property also shared by TA-2 cells. In fact, after 7–9 months of growth *in vitro*, TB6-3 cells, which were sensitive to the FCS concentration soon after their establishment *in vitro*, were afterwards able to grow in 1% FCS medium, like TA-2 cells. A further analysis of TB6-3 and TA-2 cell lines showed that their growth *in vitro* was dependent on the cell concentration since optimal growth at low serum concentration (1–2%) was achieved only when at least $10^5$ cells/well were plated; less than 50% of cell growth was observed at $10^4$ cells/well in presence of the same FCS concentration (Figure 4). This raises the possibility that A-

**Table II** Specific CTL induction by A-MuLV transformed cells

| *Effector* | *Stimulator* | *Target* | *% Specific $^{51}Cr$ release[a] at effector/target cell ratio of* 50:1 | 17:1 | 6:1 | 2:1 |
|---|---|---|---|---|---|---|
| Spleen cells from TB6-3 | TB6-3 | TB6-3 | 67 | 68 | 50 | 38 |
| immune B6 mouse[b] | TB6-3 | B6 Blasts | 0 | 0 | 0 | 0 |
| Spleen cells from TA-2 | TA-2 | TA-2 | 68 | 71 | 58 | 39 |
| immune BALB/c mouse[c] | TA-2 | BALB/c Blasts | 15 | 15 | 14 | 10 |
| Spleen cells from M-MSV | MBL-2 | TB6-1 | 43 | 22 | 14 | 9 |
| immune B6 mouse | MBL-2 | TB6-2 | 41 | 53 | 34 | 22 |
| | MBL-2 | TB6-3 | 70 | 65 | 62 | 34 |
| | MBL-2 | MBL-2 | 52 | 52 | 40 | 28 |
| Spleen cells from M-MSV | YC8 | TA-2 | 68 | 52 | 34 | 21 |
| immune BALB/c mouse | YC8 | TA-3 | 52 | 40 | 26 | 21 |
| | YC8 | YC8 | 57 | 37 | 17 | 11 |

Generation of H-2 restricted CTL against A-MuLV infected cells in BALB/c and B6 mice was obtained by injecting the mice with A-MuLV infected cells or M-MSV cell-free preparations. Normal ConA blasts were included, as controls, in each set of experiments. [a]Calculated in a 4 h incubation assay; [b] B6 mouse which did not develop tumour after injection of $10^7$ TB6-3 cells; [c]BALB/c mouse which did not develop tumour after injection of $10^3$ TA-2 cells.

MuLV transformed cell lines release a factor(s) to sustain their own growth as suggested by Sporn & Todaro (1980) for similar observations on given cell lines.

*Mitogenic activity of conditioned media*

In order to see whether A-MuLV lymphoma cells produce and secrete a mitogenic factor(s), serial dilutions of conditioned media (CM) from TB6-3 and TA-2 cell lines were tested. First, we analysed whether cells from these lines release normal lymphokines such as IL-1 and IL-2. Any attempt to induce stimulation of growth (evaluated by $^3H$-TdR incorporation) in normal, freshly isolated thymocytes (Krakauer *et al.*, 1982) and in the IL-2 dependent CTL-L cell line (Gills *et al.*, 1978) grown in the presence of CM from TB6-3 and TA-2 cultures, was unsuccessful (data not shown). On the contrary, when CM from TB6-3 and TA-2 cultures were tested on the M-MuLV lymphoma TB-5 cell line, which is not responsive to either IL-1 or IL-2 (data not shown), a significant increase in DNA synthesis was observed (Table IV).

These results support the hypothesis that A-MuLV transformed cell lines might specifically release factor(s), different from IL-1 and IL-2, which not only stimulates their own growth but is able to induce DNA synthesis in other lymphoma cells.

**Table III** *In vivo* and *in vitro* growth properties of TB6-3 cells and their derived clones

| *Cells* | *No. cells injected* | *No. pos. mice/ no. injected* | *MST[a] (days)* | *Percentage of growth[b] in presence of 1% FCS* |
|---|---|---|---|---|
| TB6-3[c] | $5\times10^6$ | 0/5 | — | 36 |
| Clone B[d] | $5\times10^6$ | 5/10 | $20\pm3$ | 86 |
| Clone C | $5\times10^6$ | 10/10 | $21\pm7$ | N.D. |
| Clone D | $5\times10^6$ | 8/10 | $20\pm9$ | 90 |
| Clone E | $5\times10^6$ | 3/3 | $59\pm5$ | 82 |
| TB6-3[e] | $5\times10^6$ | 6/8 | $20\pm5$ | 87 |

.Tumorigenicity of TB6-3 cells and their derived clones were tested by s.c. injection in adult syngeneic mice. [a]Mean survival time$\pm$1 s.d.; [b]$10^5$ cells were plated in 96 microwell plates and the percentage of growth calculated taking as 100% the incorporation of $^3H$-TdR by each cell culture in the presence of 10% FCS; [c]TB6-3 cells tested after 2 months from their establishment *in vitro*. [d]TB6-3 clones were originally derived from the non-tumorigenic TB6-3 cell lines and tested as soon as massive cultures were available; [e]TB6-3 cells tested after 8–10 months of continuous *in vitro* growth.

### Discussion

During tumour progression many genotypic (Nowell, 1986) and/or phenotypic (Nicolson, 1987) changes may occur

**Figure 3** Southern and Northern blot analysis of the non-tumorigenic TB6-3 cells and its derived tumorigenic clones. The specific v-*abl* probe was used to hybridize both EcoRI digested DNA (10 $\mu$g) extracted from primary TB6-3 cells and its derived clones (**a**) as well as total RNA (10 $\mu$g) from TB6-3 cells and its clones B and E (part **b**).

**Figure 4** Growth curves of TB6-3 and TA-2 cells as a function of FCS concentration. The cells were seeded at different cell density ($10^5$:▲——▲; $3\times10^4$:○——○; $10^4$:●——●) in microwell plates. After 48 h, 25 $\mu$Ci $^3$H-TdR were added and left overnight. The radioisotope incorporation was evaluated with a beta-counter. The percentage of growth, for each cell concentration, was calculated taking as 100% the growth of the cells in the presence of 10% FCS.

through still poorly defined mechanism(s) (Schirrmacher, 1985).

In our system various factors, inherent to both host immune response and growth potential of the cells, appear to contribute to the tumorigenic behaviour of A-MuLV lymphoma cells. These cells, like most virus transformed cells, are highly immunogenic and able to stimulate the host immunity. In B6 mice, which are known to be highly responsive to A-MuLV antigens (Risser *et al.*, 1985), the lack of *in vivo* growth of TB6-3 cells is likely to be attributable to a virus-specific cell mediated immune response rather than to the inability of cells to grow, since the same cells were able to kill all the immunosuppressed animals. However, after a long period of *in vitro* cultivation, the TB6-3 cell line as well as its clones were able to grow in immunocompetent hosts. Apparently, the malignant cells were not variants which had lost their immunogenic potential since they were still H-2 positive and behaved *in vitro* as targets for specific CTL (data not shown).

Progression to a more malignant phenotype was accompanied by loss of requirement for exogenous growth factor(s) when cells were plated at high density (Figure 4). Thus, the tumorigenic potential of A-MuLV transformed cells is related not only to the immune response of the animal but also to their growth potential. This could also be the case for

**Table IV** Growth stimulation of TB-5 cells by CM of TB6-3 and TA-2 cells in the presence of 1% FCS

| CM | Dilution | $^3$H-thymidine incorporation (cpm ± 1 s.d.) 48 h | 72 h |
|---|---|---|---|
| — | — | 4022±265 | 4756±810 |
| TB6-3 | 1:2 | 6721±168 | 11356±917 |
| | 1:4 | 5160±221 | 7396±732 |
| TA-2 | 1:2 | 8126±675 | 13752±506 |
| | 1:4 | 8001±1236 | 7702±1708 |

Growth stimulation of TB-5 cells by conditioned media (CM) of TB6-3 and TA-2 cells was tested by plating $5\times10^3$ cells/well in 96 microwell plates in the presence of 1% FCS and graded concentrations of CM. After 48 and 72 h, 25 $\mu$Ci $^3$H-TdR are added; cells were harvested after an overnight incubation and the radioisotope incorporation evaluated with a beta-counter.

TA-2 cells, (a BALB/c derived cell line), which were highly malignant despite their immunogenicity (see Tables I and II).

The cell concentration dependent fashion of growth *in vitro* suggests the involvement of an autocrine mechanism of proliferative stimulation (Sporn & Todaro, 1980). The activation of autocrine loops has been demonstrated in other spontaneous and induced haematopoietic tumours (Gordon *et al.*, 1984; Hays *et al.*, 1984; Haas *et al.*, 1986) as well as in fibroblasts transformed by A-MuLV (Twardzik *et al.*, 1982; Gebhardt *et al.*, 1986). On the contrary, three recent studies provide evidence for non-autocrine mechanisms of growth in mast cells and myeloid cells infected with A-MuLV (Cook *et al.*, 1985; Pierce *et al.*, 1985; Oliff *et al.*, 1985). In our system the finding that conditioned media (CM) from the TA-2 and TB-3 cell lines are able to stimulate the DNA synthesis of other lymphoma cell lines, suggests that these cell lines might produce and release their own growth factor(s), different from IL-1 or IL-2.

The possibility that the observed malignant progression is due to v-*abl* modifications seems unlikely since no significant differences in A-MuLV integration patterns, v-*abl* specific mRNA expression, or $P160^{gag\text{-}abl}$ production could be detected between tumorigenic and non-tumorigenic cells.

Taken together these data suggest that, following A-MuLV infection and immortalization, cells progress toward a more transformed and malignant phenotype, which apparently is not brought about by direct viral oncogene activity. This progression was relatively slow in cells of B6 haplotype and allowed us to monitor the system, whereas in BALB/c derived cells the oncogenic potential was reached in a shorter time.

It is possible that the observed malignant progression is the result of selection of a minor subpopulation already present in the primary thymic lymphoma, with a capacity for unrestricted growth. However, the genomic stability observed in v-*abl* integration and expression patterns (Figure 3A, B) argues against a clonal selection hypothesis of variants among TB6-3 cells. The *in vitro* progression could be the result of secondary changes which further alter the *in vivo* and *in vitro* growth properties of the whole cell population. The role of the *abl* oncogene in promoting these secondary events is unknown and might not be due to genetic alterations.

We wish to thank Mrs G. Miazzo for the competent technical assistance. This work was supported in part by grants from Consiglio Nazionale delle Ricerche (Progetto Finalizzato Oncologia), Associazione Italiana Ricerca sul Cancro, and Ministero Pubblica Istruzione.

### References

ABELSON, H.T. & RABSTEIN, L.S. (1970). Lymphosarcoma: Virus-induced thymic-independent disease in mice. *Cancer Res.*, **30**, 2208.

CHIECO-BIANCHI, L., COLOMBATTI, A., COLLAVO, D., SENDO, F., AOKI, T. & FISHINGER, P.J. (1974). Tumor induction by murine sarcoma virus in AKR and C58 mice. Reduction of tumor regression associated with appearance of Gross leukemia virus pseudotype. *J. Exp. Med.*, **140**, 1162.

COLLAVO, D., PARENTI, A., BIASI, G., CHIECO-BIANCHI, L. & COLOMBATTI, A. (1978). Secondary *in vitro* generation of cytolitic T lymphocytes (CTLs) in the murine sarcoma virus system. Virus-specific CTL induction across the H-2 barrier. *J. Nat. Cancer Inst.*, **61**, 885.

COOK, W.D. (1982). Rapid thymomas induced by Abelson murine leukemia virus. *Proc. Natl Acad. Sci. USA*, **79**, 2917.

COOK, W.D. (1985). Thymocyte subsets transformed by Abelson murine leukemia virus. *Mol. Cell Biol.*, **5**, 390.

COOK, W.D., METCALF, D., NICOLA, N.A., BURGESS, A.W. & WALKER, F. (1985). Malignant transformation of a growth factor-dependent myeloid cell line by Abelson virus without evidence of an autocrine mechanism. *Cell*, **41**, 677.

D'ANDREA, E., SAGGIORO, D., FLEISSNER, E. & CHIECO-BIANCHI, L. (1987). Abelson murine leukemia virus-induced thymic lymphomas: Transformation of a primitive lymphoid precursor. *J. Natl Cancer Inst.*, **79**, 189.

GEBHARDT, A., BELL, J.C. & FOULKES, J.G. (1986). Abelson transformed fibroblasts lacking the EGF receptor are not tumorigenic in nude mice. *EMBO J.*, **5**, 2191.

GILLIS, S., FERM, M.M., OU, W. & SMITH, K.A. (1978). T cell growth factor: Parameters of production and a quantitative microassay for activity. *J. Immunol.*, **120**, 2027.

GLYNN, J.L., McCOY, L. & GOLDIN, A. (1968). Cross-resistance to the transplantation of syngeneic Friend, Moloney and Rauscher virus-induced tumors. *Cancer Res.*, **28**, 434.

GOFF, S., GILBOA, E., WITTE, O.N. & BALTIMORE, D. (1980). Structure of the Abelson murine leukemia virus genome and the homologous cellular gene: Studies with cloned viral DNA. *Cell*, **22**, 777.

GORDON, J., LEY, S.C., MELAMED, M.D., AMAN, P. & HUGHES-JONES, N.C. (1984). Soluble factors required for the autostimulatory growth of B lymphoblasts immortalized by Epstein–Barr virus. *J. Exp. Med.*, **159**, 1554.

GREEN, P.L., KAEHLER, D.H. & RISSER, R. (1987). Clonal dominance and progression in Abelson murine leukemia virus lymphomagenesis. *J. Virol.*, **61**, 2192.

HAAS, M., MALLY, M.I., BOGENBERGER, J.M. & 5 others (1986). Autocrine growth and progression of murine X-ray induced T cell lymphomas. *EMBO J.*, **5**, 1175.

HAYS, E.F., GOODRUM, D., BESSHO, M., KITADA, S. & UITTENBOGAART, C.H. (1984). Leukemia-derived growth factor (non-interleukin-2) produced by murine lymphoma T-cell lines. *Proc. Natl Acad. Sci. USA*, **81**, 7807.

KRAKAUER, T., MIZEL, D. & OPPENHEIM, J.J. (1982). Independent and synergistic thymocyte proliferative activities of PHA and IL-l. *J. Immunol.*, **129**, 939.

LAEMMLI, L. (1970). Cleavage of structural proteins during the assembly of the head of bacteriophage $T_4$. *Nature*, **277**, 680.

LECLERC, J.C., GOMARD, E. & LEVY, J.P. (1972). Cell mediated reaction against tumors induced by Oncornavirus. Kinetics and specificity of immune response in murine sarcoma virus (MSV) induced tumours and transplanted lymphomas. *Int. J. Cancer*, **10**, 598.

MANIATIS, T., FRITSCH, E. & SAMBROOK, J. (1982). Molecular cloning. A laboratory manual. Cold Spring Harbor Laboratory, Cold Spring Harbor: New York.

NICOLSON, G.L. (1987). Tumor cell instability, diversification, and progression to the metastatic phenotype: From oncogene to oncofetal expression. *Cancer Res.*, **47**, 1473.

NOWELL, P.C. (1986). Mechanisms of tumor progression. *Cancer Res.*, **46**, 2203.

OLIFF, A., AGRANOVSKY, O., McKINNEY, M.D., MURTHY, V.V.V.S. & BAUSCHWITZ, R. (1985). Friend murine leukemia virus-immortalized myeloid cells are converted into tumorigenic cells lines by Abelson leukemia virus. *Proc. Natl Acad. Sci. USA*, **82**, 3306.

PIERCE, J.H., DI FIORE, P.P. & AARONSON, S.A. (1985). Neoplastic transformation of mast cells by Abelson-MuLV: Abrogation of IL-3 dependence by a nonautocrine mechanism. *Cell*, **41**, 685.

RIGBY, P.W.J., DIECKMANN, M., RHODES, C. & BERG, P. (1977). Labeling of DNA to high specific activity by nick translation. *J. Mol. Biol.*, **113**, 237.

RISSER, R. (1982). The patogenesis of Abelson virus lymphomas of the mouse. *Biochim. Biophys. Acta*, **651**, 213.

RISSER, R., KAEHLER, D. & LAMPH, W.W. (1985). Different genes control the susceptibility of mice to Moloney or Abelson leukemia viruses. *J. Virol.*, **55**, 547.

ROSENBERG, N., BALTIMORE, D. & SCHER, C.D. (1975). *In vitro* transformation of lymphoid cells by Abelson murine leukemia virus. *Proc. Natl Acad. Sci. USA*, **72**, 1932.

ROSENBERG, N. & BALTIMORE, D. (1976). A quantitative assay for transformation of bone marrow cells by Abelson murine leukemia virus. *J. Exp. Med.*, **143**, 1453.

ROWE, W.P., PUGH, W.E. & HARLEY, W.J. (1970). Plaque assay techniques for murine leukemia viruses. *Virology*, **42**, 1136.

SAGGIORO, D., DI RENZO, M.F., COMOGLIO, P.M. & CHIECO-BIANCHI, L. (1985). Different cellular substrates of Abelson leukemia virus transforming proteins kinase in murine fibroblasts and lymphocytes. In *Modern Trends in Human Leukemia VI*, Neth, R. *et al.* (eds) p. 298. Springer-Verlag: Berlin.

SAGGIORO, D., FERRACINI, R., DI RENZO, M.F., NALDINI, L., CHIECO-BIANCHI, L. & COMOGLIO, P.M. (1986). Protein phosphorylation at tyrosine residues in v-*abl* transformed mouse lymphocytes and fibroblasts. *Int. J. Cancer*, **37**, 623.

SCHER, C.D. & SIEGLER, R. (1975). Direct transformation of 3T3 cells by Abelson murine leukemia virus. *Nature*, **253**, 729.

SCHIRRMACHER, V. (1985). Cancer metastasis: Experimental approaches, theoretical concepts, and impacts for treatment strategies. *Adv. Cancer Res.*, **43**, 1.

SCOTT, M.L., DAVIS, M.M. & FEINBERG, M.B. (1986). Transformation of T-lymphoid cells by Abelson murine leukemia virus. *J. Virol.*, **59**, 434.

SEFTON, B.M., HUNTER, T. & RASCHKE, W.C. (1981). Evidence that the Abelson virus protein functions *in vitro* as a protein kinase that phosphorylates tyrosine. *Proc. Natl Acad. Sci. USA*, **78**, 1552.

SOUTHERN, E.M. (1975). Detection of specific sequences among fragments separated by gel electrophoresis. *J. Mol. Biol.*, **98**, 503.

SPORN, M.B. & TODARO, G.J. (1980). Autocrine secretion and malignant transformation of cells. *N. Engl. J. Med.*, **303**, 878.

TOWBIN, H., STAHELIN, T. & GORDON, J. (1979). Electrophoretic transfer of proteins from polyacrylamide gels to nitrocellulose sheets: Procedure and some applications. *Proc. Natl Acad. Sci. USA*, **76**, 4350.

TWARDZIK, D.R., TODARO, G.J., MARQUARDT, H., REYNOLDS, F.H. & STEPHENSON, J.R. (1982). Transformation induced by Abelson murine leukemia virus involves production of a polypeptide growth factor. *Science*, **216**, 894.

TEICH, N., BOSS, M. & DEXTER, T.M. (1979). Infection of mouse bone marrow cells with Abelson murine leukemia virus and establishment of producer cells. In *Modern Trends in Human Leukemia III*, Neth, R. *et al.* (eds) p. 487. Springer-Verlag: Berlin.

WHITLOCK, C.A., ZIEGLER, S.F. & WITTE, O.N. (1983). Progression of the transformed phenotype in clonal lines of Abelson virus-infected lymphocytes. *Mol. Cell. Biol.*, **3**, 596.

WITTE, O.N., ROSENBERG, N., PASKIND, M., SHIELDS, A. & BALTIMORE, D. (1978). Identification of an Abelson murine leukemia virus encoded protein present in transformed fibroblasts and lymphoid cells. *Proc. Natl Acad Sci. USA*, **75**, 2488.

ZINKERNAGEL, R.M. & DOHERTY, P.C. (1975). H-2 compatibility requirement for T-cell-mediated lysis of target cells infected with choriomeningitis virus. Different cytotoxic T-cell specificities are associated with structures coded for in H-2K or H-2D. *J. Exp. Med.*, **141**, 1427.